# Building health systems capable of leveraging AI: applying Paul Farmer’s 5S framework for equitable global health

**DOI:** 10.1186/s44263-025-00158-6

**Published:** 2025-05-02

**Authors:** Liam G. McCoy, Azra Bihorac, Leo Anthony Celi, Matthew Elmore, Divya Kewalramani, Teddy Kwaga, Nicole Martinez-Martin, Renata Prôa, Joel Schamroth, Jonathan D. Shaffer, Alaa Youssef, Amelia Fiske

**Affiliations:** 1 Institute for Medical Engineering & Science, Massachusetts Institute of Technology, Cambridge, MA USA; 2 Faculty of Medicine and Dentistry, University of Alberta, Edmonton, AB Canada; 3 Department of Surgery and Anesthesiology, University Of Florida College of Medicine, Gainesville, FL USA; 4 Duke Health AI Evaluation & Governance, Duke University School of Medicine, Durham, NC USA; 5 Department of Surgery, Rutgers Robert Wood Johnson Medical School, New Brunswick, NJ USA; 6 Department of Ophthalmology, Mbarara University of Science and Technology, Mbarara, Uganda; 7 Center for Biomedical Ethics, Stanford School of Medicine, Stanford, CA USA; 8 Department of Global Health and Population, Harvard School of Public Health, Boston, MA USA; 9 Faculty of Population Health Sciences, University College London, London, UK; 10 Department of Sociology, University of Vermont, Burlington, VT USA; 11 Department of Radiology, Stanford School of Medicine, Stanford, CA USA; 12 Institute of History and Ethics in Medicine, Department of Preclinical Medicine, TUM School of Medicine and Health, Technical University of Munich, Munich, Germany

**Keywords:** Artificial intelligence, Health, Equity, Health systems, Global health, Paul Farmer

## Abstract

The development of artificial intelligence (AI) applications in healthcare is often positioned as a solution to the greatest challenges facing global health. Advocates propose that AI can bridge gaps in care delivery and access, improving healthcare quality and reducing inequity, including in resource-constrained settings. A broad base of critical scholarship has highlighted important issues with healthcare AI, including algorithmic bias and inequitable and inaccurate model outputs. While such criticisms are valid, there exists a much more fundamental challenge that is often overlooked in global health policy debates: the dangerous mismatch between AI’s imagined benefits and the material realities of healthcare systems globally. AI cannot be deployed effectively or ethically in contexts lacking sufficient social and material infrastructure and resources to provide effective healthcare services. Continued investments in AI within unprepared, under-resourced contexts risk misallocating resources and potentially causing more harm than good. The article concludes by providing concrete questions to assess AI systemic capacity and socio-technical readiness in global health.

## Background

The techno-optimistic discourse surrounding artificial intelligence (AI) in healthcare positions it as a direct solution to the greatest challenges facing global health, particularly in resource-constrained settings. Advocates suggest that AI can bridge gaps in care delivery and enable access to timely, efficient, and accurate care, both improving healthcare quality and reducing inequity and cost. For example, Microsoft and the Novartis Foundation have argued that low- and middle-income countries might “leapfrog” high-income countries through AI [1], the World Bank has conjured visions of “a remote village accessing world-class healthcare thanks to AI diagnostics” [2], and a 2019 article in Forbes from Intel AI projected “annual savings of $150 billion by 2026” [3].

A broad base of critical scholarship has emerged in response, highlighting important issues with current AI technology, such as algorithmic bias and potential harm through inequitable model outputs [4–6]. In addition to these valid performance-related concerns exists a more fundamental challenge: the dangerous mismatch between AI’s imagined benefits and the material realities of healthcare systems globally. We believe that the current technology-first framing fundamentally misunderstands or willfully overlooks both the true nature of health system challenges and the material conditions required for the meaningful advance of global health equity and care quality. Indeed, the ongoing excitement about AI in healthcare represents the latest chapter in a long history of technological solutions developed in high-resource settings being promoted for deployment in resource-constrained environments, often without adequate consideration of local contexts and needs. Like previous medical technologies, AI tools risk reinforcing existing patterns of technological dependency if implemented without addressing fundamental health system requirements.

Drawing from physician-anthropologist Paul Farmer’s framework of “Staff, Stuff, Space, Systems, and Support” (5S) [7], we apply his framework for health equity to our analysis of present-day concerns around AI and global health. A lifelong advocate for health justice in Haiti, Rwanda, and other resource-constrained settings, Farmer challenged the assumption that cutting-edge medical advancements would resolve global health disparities. Instead, he insisted that high-quality care first requires investments in the material and social conditions that sustain healthcare systems.

While we are optimistic about the long-term prospects of AI tools—just as Farmer was about breakthroughs in human immunodeficiency virus (HIV) and tuberculosis (TB) treatment—the reality is that many healthcare systems lack the basic capacities needed for AI to meaningfully enhance care. Even technically perfect AI systems would fail in contexts where healthcare workers are overwhelmed, essential medicines are scarce, and basic infrastructure is unreliable. More troublingly, the current enthusiasm for AI risks diverting resources and attention from these fundamental unmet needs.

Our central thesis is that AI cannot be deployed successfully or ethically in contexts lacking sufficient social and material infrastructure and resources to provide effective healthcare services. This argument builds on theories of justice and equity in medicine [8–13] and extends beyond technical prerequisites to encompass the full scope of healthcare system requirements—from workforce development to social support structures. Building on Farmer’s commitment that it was both materially possible and ethically necessary to provide high-quality care even in parts of the world with limited resources, we argue that healthcare systems investment is a critical condition for AI development. Continued investments in AI within unprepared, under-resourced contexts risk misallocating resources and potentially exacerbating existing health inequities.

## Power, technology, and healthcare systems: a critical foundation

Technology, rather than simply a tool, is best understood as a social process, with the potential to reorder and restructure healthcare systems impacting all those who work within it. To understand why technological solutions alone cannot address the greatest challenges facing healthcare systems, we must first examine the role of power. Farmer’s critique of power dynamics in global health provides an important lens for understanding how AI implementation may mirror and amplify existing inequities. His analysis examined what he termed “pathologies of power”: how economic systems, political decisions, and social hierarchies create and perpetuate health inequities [14].

The priorities of those with technological, financial, and legal control within healthcare systems are often at odds with those of the patients they serve [15], at times pitting profit against the provision of health or promotion of community resilience. Despite good intentions that may align with patient needs, decision-makers remain removed from the lived realities of healthcare delivery by multiple forms of separation—experiential, perspectival, and often geographical. This distance can lead to solutions that, while technically or organizationally sophisticated, do not align with local contexts and patient population needs [16]. When these attempts at innovation fail to improve outcomes, a familiar pattern emerges: a reliance on what Farmer termed “immodest claims of causality.” Rather than recognizing the structural barriers behind AI technology failures, these claims often attribute shortcomings in outcomes to local cultural practices or individual behaviors. This perspective deflects accountability from systemic issues, privileging those in positions of power—those who design and market these technologies—by framing structural problems as cultural or behavioral failings [17].

This disconnect becomes particularly acute in the context of AI development and deployment. AI systems are typically developed in wealthy academic medical centers and technology companies, environments that bear little resemblance to the resource-constrained settings where they may be deployed [18]. The resulting tools often embed assumptions about available resources, workflow patterns, and infrastructure that simply do not hold true in many healthcare contexts. A clear example of this occurred when a Google diabetic retinopathy model which was highly successful in initial testing failed in deployment in Thailand due to issues with provider training, internet availability, clinic lighting quality, and patient trust [19].

Furthermore, the process of AI development itself tends to concentrate power in the hands of those already most distant from care delivery. Data flows predominantly from resource-constrained settings to wealthy institutions, while control over the resulting tools remains firmly in the hands of technical experts and technology companies. This dynamic risks creating a new form of technological dependency, where healthcare systems become reliant on AI tools they cannot meaningfully influence or adapt to local needs [20–23].

The current enthusiasm for AI in global health must be considered against the backdrop of persistent underinvestment in basic health infrastructure [24]. Many of the same governmental institutions promoting AI solutions (including the World Bank and International Monetary Fund (IMF)) have historically supported policies—from structural adjustment programs to intellectual property regimes—that weakened public health systems in low- and middle-income countries (LMICs) [25–31]. For example, loans conditionality administered by the IMF, World Bank, and African Development Bank (AfDB) and others forced ministries of health to implement a dramatic expansion of patient-level user fees (euphemistically called “cost-sharing”) which had the effect of both reducing overall “fiscal space” and health systems revenue as well as “greatly reduc[ing] access to even the most rudimentary health services for the poor” [32].

Given Farmer’s searing critiques of how global economic policies perpetuated epidemics like HIV and multi-drug–resistant tuberculosis (MDR-TB) [14, 17], we are compelled to ask: who benefits from pushing an AI-first narrative for healthcare, and does this paradigm truly serve the interests of the most disadvantaged patients? Building on these critical foundations, we apply Farmer’s 5S framework to the present-day challenge of building healthcare systems capable of leveraging AI. Throughout our analysis of the specific requirements for AI-ready healthcare systems, we maintain a focus on power relations and sociotechnical dynamics. Each component of the framework—Staff, Stuff, Space, Systems, and Support—involves not just technical capabilities but questions of control, access, and equity that must be addressed before meaningful AI implementation can be considered [33]. Without these components, we risk building AI systems that serve only a privileged few, worsening health inequities by diverting resources from fundamental healthcare services to technologies that, while sophisticated in some settings, are poorly suited to the realities of others. The application of the 5S framework to the use of AI in global health is an argument that healthcare systems investment needs to be at the forefront of any aspirations for using AI for good.

## Staff: healthcare workers must be supported before AI can be considered

Farmer placed “Staff” at the forefront of his framework because healthcare systems are fundamentally human enterprises—networks of trained professionals working to provide care for their patients and communities. The “availability, accessibility, acceptability and quality” of healthcare staff determines a system’s capacity to deliver effective and high-quality care [34, 35].

Globally, staff shortages are endemic, with clinical professionals often functioning in complex, overstretched environments where demand outpaces supply. These workforce challenges follow a socioeconomic gradient, with the most critical shortages in the poorest regions and nations—a key element of the inverse care law [36, 37]. This stark reality raises a crucial question: what are the consequences of implementing AI in healthcare systems without addressing the critical need for essential human capital to provide quality care?

The relationship between healthcare staff and AI requires careful consideration [38]. The narrative of AI “empowering” clinicians often masks a more complex reality where technological implementation serves to restructure clinical practice and redistribute power within healthcare organizations. Rather than simply augmenting clinical capabilities, technological implementation may even shift control away from frontline healthcare workers toward administrators, technology vendors, and distant institutions that design and control these systems.

Previous waves of healthcare technology have sometimes contributed to worker alienation, erosion of professional autonomy, and displacement of clinical judgment [39–41]. This can limit the ability to provide high-quality medical care, making it more difficult for healthcare professionals to promote the wellbeing of patients or to prevent harm from occurring. The current wave of AI implementation risks further ignoring such cautionary tales, particularly given the unique power dynamics of AI, as workers may be pressured to adapt their practice to accommodate tools and systems that they have had little involvement in developing or deploying while carrying the liability for their use. These lessons from history suggest that strengthening and empowering the healthcare workforce must precede, not follow, technological advancement.

Building strong healthcare workforces requires sustained investment in several key areas:

1. Recruitment and retention of healthcare professionals at all levels to create a dynamic, diverse, and resilient workforce [42].
2. Fair compensation and working conditions that recognize healthcare work as skilled labor, supported by organization policies and legal protections [43].
3. Organizational cultures that value and incorporate frontline worker perspectives.
4. Quality medical education and health professional training along with development pathways that support career growth and facilitate the development of expertise in working with technologies, with protected time for staff to engage in system improvement and innovation [44, 45].
5. Leadership that recognizes the need for careful co-design of technology with staff as well as rigorous monitoring and iterative evaluation of the impact of new technologies on care delivery.

When considering AI implementation, healthcare systems must demonstrate their ability to recruit, develop, retain, and support human healthcare workers. Systems struggling with basic staffing should prioritize workforce development over AI investment. In addition, in well-resourced settings, AI initiatives should be evaluated using broad metrics that include their impact on staff wellbeing and professional autonomy (such as the ability to override AI when clinically appropriate). Only with a well-prepared, appropriately supported workforce can healthcare systems create conditions where AI tools might eventually enhance rather than undermine care delivery [46].

## Stuff: basic resources and infrastructure must precede technological investment

“Stuff” in Farmer’s framework refers to the essential materials and tools required for effective care delivery. While discussions of AI in healthcare often focus on sophisticated computational infrastructure, this misses a crucial point: many healthcare systems still struggle to maintain reliable access to basic medicines, supplies, and equipment. This reality demands we reconsider the relative priority of AI investment against fundamental material needs [47].

Technological sophistication cannot overcome fundamental material scarcity. AI tools might suggest optimal treatment plans, but these become meaningless without reliable access to the recommended interventions. Consider an AI system that perfectly predicts sepsis: without consistent access to antibiotics, fluid resuscitation equipment, and monitoring devices, such predictions cannot translate into improved patient outcomes.

The capital expenditures required to sustain AI performance must be weighed against the investments necessary to meet these other needs. More than half of healthcare facilities in sub-Saharan Africa continue to lack reliable electricity [48]—it is unethical to argue that servers should be powered over refrigerators, ventilators, and incubators because to do so would be to privilege care that might potentially improve care for a select few in the name of life-sustaining care for many. In impoverished settings, even modest AI expenditures represent significant opportunity costs: the price of a single month’s cloud computing services could instead purchase essential medicines, diagnostic equipment, or basic medical supplies that directly impact patient care.

These material challenges cannot be separated from broader structural factors which shape access to essential healthcare resources. Global intellectual property regimes and pharmaceutical pricing structures often render life-saving medications unaffordable precisely where they are most needed. The HIV/AIDS crisis provided a stark illustration of how patent laws and profit-driven drug development can create an artificial scarcity of essential medicines, and these same structural barriers continue to limit access to essential medications in many healthcare systems today [29–31].

Any discussion of healthcare “stuff” must recognize these power dynamics—the issue is often not technological capability but political and economic structures that restrict access to existing resources. The same power structures that create artificial scarcity of basic medicines now shape access to AI technologies, raising questions about who truly benefits from technological advancement in healthcare.

To address these challenges and strengthen material resources in healthcare systems, the field must focus on:

1. Building robust supply chains for essential medicines and supplies.
2. Developing local manufacturing capacity where feasible.
3. Establishing effective maintenance programs for existing equipment.
4. Creating reliable inventory management systems.
5. Ensuring consistent access to basic utilities (electricity, water, internet).
6. Addressing global economic and structural barriers to material access.

These improvements are valuable independent of any potential AI implementation. Reliable access to essential medicines and functional equipment directly improves patient care. Strong supply chains and inventory systems reduce waste and stockouts. Local manufacturing capacity builds system resilience and contributes to economic development.

If AI tools eventually prove valuable, these same material strengthening efforts would enable their implementation—but this should be seen as a secondary benefit rather than a primary justification. Only when healthcare systems can consistently provide essential medicines, maintain basic equipment, and ensure reliable access to fundamental utilities will they be able to benefit from investments in advanced technological infrastructure.

## Spaces: physical healthcare infrastructure cannot be leapfrogged by digital solutions

A key component of Farmer’s framework emphasizes “safe, appropriate spaces with capacity to serve patients” [7]. Numerous regions of the world lack adequate healthcare spaces. Even where such facilities do exist, many communities face significant barriers to accessing them due to geographic distance, inadequate transportation infrastructure, or social and cultural barriers [49]. These spatial inequities often reflect and reinforce broader patterns of marginalization. While AI enthusiasts sometimes suggest that digital health can transcend physical barriers to access, this optimism overlooks a fundamental reality: the vast majority of healthcare interventions—from preventive care to emergency services—require physical spaces for delivery [50, 51].

The physical infrastructure challenges facing healthcare systems are both severe and multifaceted. Many healthcare facilities lack reliable electricity, clean water, or adequate infection control infrastructure. Even basic requirements for dignified care delivery—private examination rooms, sterile operating theaters, and secure pharmaceutical storage—remain absent in numerous settings [52]. These deficiencies create immediate risks to patient safety and care quality while also limiting the types of services that can be safely provided. The resource gradients are stark: while some regions struggle to maintain basic clinic buildings, others are constructing “hospitals of the future” with sophisticated environmental controls and integrated digital systems.

Against this backdrop of widespread infrastructure deficits, discussions of digital spaces and AI implementation require careful consideration. Digital spaces must be understood as extensions of, not replacements for, physical healthcare infrastructure. Digital security and privacy considerations become particularly acute in settings where basic infrastructure is precarious. Many healthcare systems in LMICs face challenges with reliable internet connectivity, data storage capabilities, and cybersecurity infrastructure. These limitations cannot be solved through technological solutions alone—they require sustained investment in both physical and digital infrastructure [53]. Foremost priorities should include the following:

1. Investing in well-equipped, clean, and adequately staffed medical facilities that can support both routine and specialized care.
2. Addressing transportation barriers and geographic inequities by establishing or strengthening infrastructure in underserved regions.
3. Ensuring that healthcare spaces have stable access to essential utilities including electricity, clean water, and sanitation.
4. Developing digital infrastructure in tandem with physical spaces (e.g., reliable internet access, secure data storage, and IT support systems) while recognizing that digital solutions cannot replace physical facilities.

The implications for health system strengthening are clear: healthcare spaces must be evaluated based on their direct contribution to patient care and accessibility, not their potential to enable future technological advancement. Only when healthcare systems can consistently provide safe, appropriate physical spaces are they prepared to consider significant investments in digital infrastructure. This ordering of priorities reflects both practical necessity and ethical imperative: commitments to ethical values such as equity or justice [8–13] in AI require that the fundamental right to access appropriate healthcare spaces precede considerations of technological enhancement.

## Systems: strong healthcare governance as a foundation for technological innovation

At their core, healthcare systems are interconnected webs of relationships, power structures, and institutional processes. Applying Farmer’s system perspective, we see that while wealthy institutions debate sophisticated AI governance frameworks, many healthcare systems (including in wealthy nations) still struggle with basic operational challenges—from unreliable supply chains to fragmented patient records to weak regulatory oversight to inadequate quality control mechanisms. These fundamental systemic weaknesses cannot be bypassed by technological solutions alone; indeed, attempting to layer AI systems over unstable institutional foundations risks exacerbating existing problems.

The question of AI integration thus becomes secondary to the more fundamental challenge of building robust, equitable systemic structures. This requires not just technical capacity but genuine democratization of healthcare governance, with meaningful inclusion of patient and healthcare worker voices in system design and operation [54].

First, governance must precede technological implementation. Many healthcare systems lack resilient governance structures for existing technologies, let alone AI. Clear frameworks for oversight, accountability, and ethical decision-making premised on principles of justice and equity are essential for all aspects of healthcare delivery—from basic medical procedures to advanced technologies.

Second, local leadership and expertise must be centered. The principle of “nothing about us without us” needs to be applied not just to AI development but to all aspects of healthcare system design and operation [55]. Local healthcare workers and communities understand the systemic constraints and opportunities within their contexts in ways that external actors cannot.

Third, financing models must reflect systemic priorities. In resource-constrained settings, investments must be carefully evaluated against fundamental healthcare needs. A systems perspective demands an honest assessment of trade-offs: how do potential AI implementation costs compare to investments in basic healthcare infrastructure, essential medicines, or workforce development? What are the ethical trade-offs in obligations to provide high-quality healthcare that promotes individual and collective well-being?

These resource allocation decisions represent profound ethical dilemmas, forcing stakeholders to weigh competing moral obligations: the obligation to provide basic care to the maximum number of people versus investing in technologies that might improve care quality for a subset of patients. Allocating scarce resources to AI implementation before ensuring universal access to essential services risks violating principles of distributive justice and exacerbating health inequities. Similarly, the principle of non-maleficence requires us to avoid the harm that might come from diverting resources from life-saving interventions to technological systems that may not function effectively in under-resourced environments.

Finally, IT infrastructure must be understood as a systemic issue rather than merely a technical one. Questions of data governance, privacy, and security are fundamentally about power relationships—who controls health information, how it is used, and who benefits from its analysis [53]. Building solid IT systems may improve care coordination, enable outcome tracking, and support evidence-based decision making, even in the absence of AI tools.

AI can only support healthcare systems with a fairly mature capacity for governance, strategic business decisions, and IT infrastructure. Key steps include the following:

1. Establishing oversight frameworks to manage AI co-development, purchasing and implementation, ensuring ethical, and transparent decision making.
2. Centering local leadership and community participation to align AI adoption with real-world healthcare needs and system capacities.
3. Developing sustainable financial models that align AI investments with health system priorities and prevent resource diversion from essential services.
4. Strengthening health information systems with reliable records, secure data-sharing, and robust cybersecurity.
5. Conducting regular audits and evaluations to assess AI readiness, effectiveness, and equity impacts.

The focus on AI in healthcare risks creating a narrative where these systemic improvements are viewed merely as prerequisites for technological advancement. This framing gets the relationship backward. Strong governance, local leadership, sustainable financing, and reliable IT infrastructure are essential components of effective healthcare systems in their own right. When healthcare systems can demonstrate these capabilities, they are not merely “AI-ready”—they are fulfilling their fundamental purpose of providing quality care to their communities. Only in such contexts should AI implementation even begin to enter strategic discussions.

## Support: social infrastructure determines healthcare success more than technology

The “Support” component of Farmer’s 5S framework focuses on the social and economic ecosystem essential for effective healthcare delivery [7]. This encompasses everything from social safety nets to working conditions, from food security to transportation systems. Those in well-resourced settings may view these factors as outside of the purview of the healthcare system, yet they often have the greatest impact on patients’ ability to benefit from care. Healthcare projects that ignore these factors often fail to achieve impact, and this remains true for AI.

Consider an AI system designed to optimize medication adherence: even if technically perfect, it cannot succeed where patients cannot afford prescribed medications, lack reliable transportation to pharmacies, or work multiple jobs that make regular medication schedules impossible. AI systems trained on data from populations with stable housing and food security may make inappropriate or even harmful recommendations for communities where these basic needs remain unmet. The technology-first mindset fundamentally misunderstands how social conditions determine healthcare outcomes.

This lens further expands the scope of the trade-offs we have discussed throughout this article. The cost of AI-related investments must be weighed not only against other healthcare expenditures but also against the impact of measures such as providing food or direct financial support to patients and their families.

Effectively providing this support requires deep engagement with and understanding of communities and their needs. It requires fundamental investment into building trust between historically marginalized communities and the health and social systems that purport to support them but have often failed to do so.

Healthcare systems must work with other elements of the social care infrastructure to develop and maintain:

1. Robust social safety nets that address fundamental determinants of health outcomes. This includes not just direct healthcare support but comprehensive programs addressing food security, housing stability, and economic well-being.
2. Real community partnerships with foundations of trust and sustained commitment, particularly with communities that have experienced historical exploitation by healthcare institutions
3. Financial support which extends beyond direct medical costs to include transportation, childcare, and lost wages in order to make care truly accessible.

Healthcare outcomes are determined far more by social conditions than by technological sophistication [56]. A community with strong social support systems but basic medical technology will generally achieve better health outcomes than one with advanced AI systems but inadequate social infrastructure. While AI might eventually help optimize resource distribution or identify high-risk individuals, such applications should be seen as potential future refinements to already-strong social support systems, not as solutions to fundamental social challenges.

## Discussion

The promise of AI in healthcare has generated significant enthusiasm, with proponents suggesting that AI can bridge gaps in care delivery, particularly in resource-constrained settings. In this viewpoint, we are not arguing against the use and development of AI technologies in global health; on the contrary, there are many opportunities to address critical health concerns using technological advances. However, our analysis reveals a stark reality: most healthcare systems are nowhere near ready for meaningful AI implementation. This disconnect is evident in what has been termed the “implementation gap”—the continued investment in AI without corresponding improvements in healthcare outcomes [57–59].

While much of the discourse on AI in healthcare focuses on algorithmic performance and potential biases, here, we draw attention to a more fundamental problem: many healthcare systems still struggle to provide basic care, lacking essential staffing, supplies, and infrastructure [60]. In this context, prioritizing AI implementation is not merely premature—it risks causing negative outcomes across multiple domains and actively undermining healthcare delivery by misdirecting scarce resources.

To guide healthcare leaders in evaluating their readiness for technological advancement, we propose the following questions structured around the 5S framework (Table 1). These questions are designed to evaluate whether a healthcare system has achieved sufficient strength in its fundamental components that it could responsibly consider AI implementation. These questions are designed for multiple stakeholders involved in healthcare AI decisions. Healthcare system leaders (including both executives and clinical leadership) should use these questions to assess organizational readiness; AI developers should consider these factors when designing and marketing solutions, funders and policymakers should incorporate these criteria into investment and regulatory decisions, and frontline clinicians can use these questions to advocate for necessary system improvements before AI implementation. Importantly, answering “no” to these questions should not be seen as barriers to be overcome on the path to AI implementation, but rather as crucial areas requiring investment in their own right. Only when healthcare systems can demonstrate sustainable capabilities across all 5S dimensions should AI implementation begin to enter strategic discussions.

**Table 1:** Readiness questions for healthcare systems, developers, and funders considering AI investment

Priority	Readiness questions
Staff	• Does the healthcare system employ a diverse range of trained professionals in sufficient numbers? Are these professionals compensated equitably across all levels of hierarchy? • Does the healthcare system have standardized, dedicated time and resources for training and upskilling clinical staff? • How is the healthcare system planning for anticipated changes in staffing needs, roles, and skill requirements over a clear time horizon? To what extent is the planning process collaborative, allowing for meaningful engagement of the clinical staff? • Has leadership conducted a systems-level analysis to identify who may benefit (financially, socially, politically, or otherwise) from the introduction of new health technologies, and who might be disadvantaged? Are there plans to track, measure, and mitigate the impact (both intended and unintended) of these technologies on staff? • When considering AI tools procurement, where are the technologies sourced from? Are they co-created with relevant local healthcare staff, and is training inclusive of all relevant personnel? Are global or regional differences considered in training, implementation, and oversight? • What mechanism does the healthcare system have to ensure the transparency and intelligibility of algorithmic outputs? How will healthcare professionals be trained and empowered to review, question, and challenge algorithmic decisions?
Stuff	• Does the healthcare system have the essential materials and tools needed to provide high-quality care for all patients? • Does the healthcare system have the essential technical materials, such as consistent electricity, internet access, computer hardware and more? • Does the healthcare system have the means to share and integrate data with other local or regional healthcare systems? • Are the tools for providing care integrated into an automated system to collect and analyze data? • Does the healthcare system have a robust data infrastructure to provide secure, scalable data storage of large volumes of health data? • Does the healthcare system have privacy and cybersecurity protocols in place? • Does the healthcare system have an efficient supply chain and resource management plans? • Does the healthcare system have local or regional level manufacturing of medical supplies?
Spaces	• Does the healthcare system have equipped, clean, physical facilities for providing care for comprehensive medical services? • Does the healthcare system have safe digital spaces, with reliable internet connectivity and cloud access? • Does the healthcare system have protocols in place to ensure the safety of both physical spaces (sufficient privacy, supportive services e.g. for those afraid of accessing care or dealing with stigma or in need of additional services) and digital spaces (data protection, data privacy, encryption and authentication protocols, regular audits)?
Systems	• Have the set of systems that comprise and are comprised by your healthcare system been documented and mapped (including social relationships, political leadership, governance, IT and resources, as well as the local histories that contribute to the ongoing production of systemic injustices and disparities)? • Does the healthcare system regularly conduct audits and analyses at micro, meso, and macro levels to track health outcomes, quality of service, and patient experience? • Is leadership clear about where the greatest staffing needs are to improve care delivery? • Does the healthcare system have clear, established, fit-for-purpose processes and standards for implementing new healthcare technologies? Are these rigorous and adaptable to new technologies like AI (e.g., including steps for local validation and monitoring)? • Is there a clear financing plan for the introduction of new technologies, including an analysis of areas within the healthcare system that may face reduced funding or be deprioritized as a result?
Support	• Does the healthcare system have processes in place to support patients and community members comprehensively? • Are there systems to provide social support—such as food, housing, education, employment—for staff, patients, and community members? • Are there regional, local, and system-wide mechanisms in place to reduce poverty and support vulnerable and marginalized groups? • Is the healthcare system actively implementing initiatives to address disparities in health outcomes?

The implications for health system strengthening initiatives are twofold. First, we must recognize that most healthcare systems require sustained investment in basic infrastructure and capabilities before AI implementation can be meaningfully considered. Second, these systemic improvements should be evaluated based on their immediate benefits for healthcare delivery, not only their potential to enable future AI implementation. If AI technologies ultimately prove less transformative than hoped, investments in these systemic improvements will still yield substantial benefits for healthcare delivery and patient outcomes.

It is also important to recognize that these issues are not merely limited to LMICs or even low-resource contexts in high-income countries (HIC). The healthcare system of the USA, for example, is notorious for issues of system complexity leading to inaccessibility and cost challenges leading to a lack of support [61]. Further, there are and will continue to be LMIC health systems—such as that of Kerala, India [62] —which act as exemplars for their embodiment of 5S principles. We remain hopeful that AI may have much to offer in furthering health system development when built upon and alongside strong foundations, and we expect that examples of effective deployment may occur in a variety of countries.

By privileging health system readiness, we are not calling to “halt” all AI technologies, nor insist that AI technologies are exclusively employed under “ideal” circumstances in global health (something that is not even true in many healthcare systems in HIC). Rather, applying the 5S analysis to AI in global health shows that without the strong foundations of a robust, resilient healthcare system, AI will not fulfill its promises for improved healthcare access, outcomes, or reductions in costs or inequities. Following Farmer’s lead, healthcare systems readiness is an argument that it is both materially possible and ethically necessary to invest in healthcare systems in global health—both for their own sake and as a critical condition for AI development. Further, there is no single, universal threshold for readiness. Rather, we believe that readiness must be assessed in a context-dependent fashion specific to the health system and technology at hand. The readiness questions in Table 1 are designed to encourage context-based assessments.

As we move forward, it is crucial that funders, policymakers, and healthcare leaders resist the allure of technological solutions and instead prioritize the difficult, complex work of building robust health systems. This means making difficult but necessary decisions to invest in infrastructure and workforce development rather than the newest AI tools. Those looking to develop AI in healthcare, including research labs and technology companies, should consider the role they might play in supporting this development and contributing to building health systems capable of leveraging these technologies. Investing in the work required to build resilient health systems will enable technological innovations that truly help to improve the health of all, including efforts that focus on the complementarities of human expertise and AI [63, 64], and engage co-design approaches to technology development to ensure that local healthcare workers and community needs are at the center of these developments. Such approaches could help to integrate AI tools in a way that bolsters, rather than substitutes, healthcare delivery. Doing so can foster collaboration and decision-making among local healthcare workers while also investing in important historical, political, and socio-cultural matters that bear on medical practice [65].

## Conclusions

The allure of AI in healthcare reflects several persistent patterns in global health: the embrace of the new shiny object at the expense of less glamorous but essential investments in the 5S’s, comfort in the simplicity of a narrow technological solution for what are fundamentally complex system challenges, and finally, the latest iteration of novel solutions that are developed in the global north and imported to LMICs. The current hype around healthcare AI represents more than just misplaced optimism—it risks actively reinforcing global health inequities by diverting resources and attention from fundamental systemic needs, while consolidating control over healthcare delivery in the hands of technology providers. This dynamic mirrors historical patterns where technological “solutions” have, at times, served to maintain the status quo rather than truly improve health inequality.

Applying the 5S analysis means critically assessing healthcare systems readiness (Table 1). In addition, this work needs to be accompanied by healthcare policies that take a holistic approach to AI technologies, recognizing that questions of AI investment, development, and governance all are deeply entwined with building resilient healthcare systems. Building from this, funders of AI technologies need to consider the implementation context as an integral part of technology development. To ensure sustainable implementation, funders of healthcare AI should adopt a balanced investment approach: for every dollar spent on technology development, a proportional investment should strengthen the foundational healthcare system components that will ultimately determine AI’s success.

As Farmer’s work consistently demonstrated, meaningful improvements in health outcomes require political commitment and sustained investment in basic healthcare infrastructure to address underlying inequities. Until healthcare systems can demonstrate sustainable capabilities across all dimensions of the 5S framework—supporting skilled staff, maintaining essential supplies, providing safe spaces, implementing strong governance, and addressing social determinants of health—AI implementation runs the risk of not just being premature but potentially harmful. The measure of success in healthcare should not be the sophistication of our technology, but the consistent delivery of quality care to all who need it.

## Data Availability

No datasets were generated or analysed during the current study.
